# A new widespread subclass of carbonic anhydrase in marine phytoplankton

**DOI:** 10.1038/s41396-019-0426-8

**Published:** 2019-04-25

**Authors:** Erik L. Jensen, Romain Clement, Artemis Kosta, Stephen C. Maberly, Brigitte Gontero

**Affiliations:** 1 Aix Marseille Univ, CNRS, BIP, UMR 7281, IMM, FR3479, 31 Chemin J. Aiguier, 13402 Marseille Cedex 20, France; 2 Microscopy Core Facility, Aix Marseille Univ, CNRS, IMM, FR3479, 31 Chemin J. Aiguier, 13402 Marseille Cedex 20, France; 3 Lake Ecosystems Group, Centre for Ecology & Hydrology, Lancaster Environment Centre, Library Avenue, Bailrigg, Lancaster, LA1 4AP UK

**Keywords:** Water microbiology, Environmental chemistry

## Abstract

Most aquatic photoautotrophs depend on CO_2_-concentrating mechanisms (CCMs) to maintain productivity at ambient concentrations of CO_2_, and carbonic anhydrase (CA) plays a key role in these processes. Here we present different lines of evidence showing that the protein LCIP63, identified in the marine diatom *Thalassiosira pseudonana*, is a CA. However, sequence analysis showed that it has a low identity with any known CA and therefore belongs to a new subclass that we designate as iota-CA. Moreover, LCIP63 unusually prefers Mn^2+^ to Zn^2+^ as a cofactor, which is potentially of ecological relevance since Mn^2+^ is more abundant than Zn^2+^ in the ocean. LCIP63 is located in the chloroplast and only expressed at low concentrations of CO_2_. When overexpressed using biolistic transformation, the rate of photosynthesis at limiting concentrations of dissolved inorganic carbon increased, confirming its role in the CCM. LCIP63 homologs are present in the five other sequenced diatoms and in other algae, bacteria, and archaea. Thus LCIP63 is phylogenetically widespread but overlooked. Analysis of the Tara Oceans database confirmed this and showed that LCIP63 is widely distributed in marine environments and is therefore likely to play an important role in global biogeochemical carbon cycling.

## Introduction

Diatoms are photosynthetic microorganisms that occur in most aquatic environments [1, 2]. Their ecological success relies on a distinctive metabolism [3–5] that results from their complex evolutionary history and consequently diatom genomes contain genes from archaea, bacteria, animals, green and red algae, and the eukaryote host [4]. Marine diatoms contribute up to 20% of the global primary productivity [2] and rely on CO_2_-concentrating mechanism (CCMs) in order to maintain productivity at the low concentration of CO_2_ found in the oceans [6]. Diatom CCMs involve the active transport of CO_2_ and/or HCO_3_^−^, and, less certainly, C4 photosynthesis [7–9], and the enzyme carbonic anhydrase (CA; EC 4.2.1.1) that catalyzes the rapid interconversion of HCO_3_^−^ and CO_2_.

CA is found throughout the tree of life and takes part in numerous processes [10]. Seven distinct CA subclasses have been described to date (α, β, γ, δ, ζ, η, and θ-CAs) [11]. Some CAs are constitutively expressed in diatoms [12], but they are also an important component of CCMs where they are among the most highly upregulated proteins in cells grown at low- versus high-concentrations of CO_2_ [13]. CAs are all metalloenzymes that commonly use zinc (Zn^2+^) as a cofactor, however, in the diatom *Thalassiosira weissflogii*, the ζ-CA (CDCA) and the δ-CA (TWCA1) are able to replace Zn^2+^ with cadmium (Cd^2+^) and with cobalt (Co^2+^), respectively [14–16]. Six CA subclasses have been found in diatom genomes, and their subcellular localization has been predicted in model species [12, 17].

The centric coastal diatom, *Thalassiosira pseudonana* was the first diatom to have its genome sequenced and has been widely studied [18]. In this species, there is physiological evidence for a biophysical CCM and genes of the solute carrier SLC4 family are present in its genome [19]. In addition, thirteen putative CAs from four different subclasses have been reported in this species, located in the periplasmic space, the cytoplasm, the periplastidal compartment, and the chloroplast stroma [12].

Recently, a protein of unknown function, LCIP63 (*Low-CO*_*2*_*-inducible protein of* 63 kDa, Joint Genome Institute protein ID 264181) was found in *T*. *pseudonana* [13]. LCIP63 is rapidly and substantially upregulated in cells at low CO_2_ concentration [13, 20], but downregulated when cells are limited by phosphorus, nitrogen, silicon, or iron [13], a response consistent with a reduced demand for carbon. We hypothesized that LCIP63 could be a previously undescribed CA since, like other CAs, it is upregulated at low CO_2_ concentration [13]. Moreover, the amino acid sequence of LCIP63 bears an endoplasmic reticulum signal peptide and a chloroplast transit peptide suggesting that it is located in the chloroplast. Therefore, it could play a role in the CCM of *T*. *pseudonana*.

Here we show, using in vivo and in vitro approaches that LCIP63 is indeed a new subclass of CA, an important chloroplastic component of the CCM in *T*. *pseudonana*, uses Mn^2+^ as a cofactor and is taxonomically and geographically widespread.

## Methods

### Cells and culture conditions

The sequenced *T. pseudonana* clone (CCMP1335) was obtained from the Culture Collection of Algae and Protozoa and grown in artificial sea water supplemented with half ‘F’ Guillard’s medium plus silicon (F/2 + Si) at 18 °C under continuous illumination at ~50 µmol photon m^−2^ s^−1^ (400–700 nm). To induce LCIP63 expression, cultures were grown at 20,000 ppm (2%) CO_2_ for 96 h then transferred to 50 ppm (0.005%) for 24 h since LCIP63 is rapidly induced from 3 h after this transfer [13].

### Data mining for LCIP63 sequence

Sequences similar to LCIP63 from *T*. *pseudonana* were searched using the “protein BLAST” software from the National Center for Biotechnology Information (NCBI) website (https://blast.ncbi.nlm.nih.gov/Blast.cgi) using the default settings. Searches were restricted to diatoms for diatom LCIP63 homologs. When searching for other organisms, diatoms were excluded. CA sequences from the different subclasses were also searched through the NCBI website. Sequences were selected where the *E*-value was less than 1.0 E^−24^. The neighbor joining phylogenetic tree was created using the bootstrap test of phylogeny option available in the Mega6 software [21]. 1000 pseudoreplicates were applied for bootstrapping.

The LCIP63 protein sequence was queried against the Ocean Gene Atlas webserver (http://tara-oceans.mio.osupytheas.fr/ocean-gene-atlas/) [22] using the Tara Oceans Microbiome Reference Gene Catalog version 1 to search for prokaryote homologs and the Marine Atlas of Tara Oceans Unigenes version 1 Metatranscriptomes for eukaryotes. Threshold *E*-values are given in the main text.

### Heterologous expression of LCIP63

The nucleotide sequence of LCIP63 without introns and the signal peptide sequence was produced synthetically (GeneCust, Ellange, Luxembourg) and inserted between *NdeI* and *XhoI* restriction sites of a pET-28a+vector so that the protein was fused to a His-tag on its N-terminus. The sequence was optimized according to *E*. *coli* codon usage to enhance expression. Variants containing two and three repeated domains were synthesized using this procedure. The gene of LCIP63 was cloned and overexpressed in the *E*. *coli* strain BL21-C41(DE3). Positive clones were selected using kanamycin resistance (33 µg ml^−1^). Expression of recombinant LCIP63 in *E*. *coli* was induced by 1 mM IPTG at 37 °C for 5 h. The pellet was collected by centrifugation at 3500 *g* for 20 min at 4 °C (Allegra® X-15R Centrifuge, Beckman Coulter).

### LCIP63 purification and size-exclusion chromatography

The pellets of *E*. *coli* cells were broken using an ultrasonicator (Sonics & Materials Inc, Vibracell, Bioblock, Danbury, CT, USA) in a buffer: 50 mM sodium phosphate, 10 mM imidazole, and 50 mM NaCl (pH 8), plus lysozyme (final concentration: 40 µg ml^−1^) and a protease inhibitor cocktail (Sigma®; Concentrations: 2 mM AEBSF, 0.3 μM Aprotinin, 116 μM Bestatin, 14 μM E-64, 1 μM Leupeptin, and 1 mM EDTA). The lysate was centrifuged at 16,000 *g* for 30 min at 4 °C (Sigma® 2-16KC Centrifuge; Rotor 12132-H, Fisher Bioblock Scientific) and the supernatant loaded onto a Ni-NTA column (height 6 cm and diameter 1.5 cm) and washed at least four times with the column volume with the buffer. The column was then washed at least 4 times with the column volume with buffer containing 0.15 M imidazole, followed by a second step with buffer containing 0.35 M imidazole to elute LCIP63. These fractions were pooled, concentrated, and dialyzed with 20 mM Tris, 50 mM NaCl (pH 8). Glycerol (10% final concentration) was added prior to size-exclusion chromatography and for storage at −80 °C.

Proteins eluted from NiNTA and containing LCIP63 were loaded onto a HiLoad^TM^ 16/60 Superdex^TM^ 200 prep grade column (S200; GE Healthcare) pre-equilibrated with 20 mM Tris, 50 mM NaCl (pH 8). Proteins were followed by measuring absorbance at 280 nm. Fractions were collected separately and concentrated using a Vivaspin20 ultrafiltration tube (30,000 MWCO; Sartorius). The same procedure was followed for LCIP63 containing three and two repeated domains but the NaCl concentration was changed to 0.15 M and 0.2 M for each form, respectively, in all buffers.

### Carbonic anhydrase and esterase activity

CA activity was measured at 3 °C using a Perkin Elmer spectrophotometer (PTP-6 Peltier System), using the Wilbur and Anderson method [23], modified as described [24]. The reaction mixture comprised 1600 µl of buffer 25 mM Tris, pH 9.1, 6.4 µM bromothymol blue, plus 400 µl of CO_2_-saturated water. The reduction in pH was followed spectrophotometrically by measuring the change in absorbance at 620 nm. Enzyme activity was calculated as Wilbur-Anderson arbitrary units (WAU) mg^−1^ protein after adding 10–20 µg of sample, in comparison to a blank. To determine metal ions requirements, samples were kept in 20 mM Tris, NaCl (50, 150, or 200 mM), pH 8, with or without 5 mM EDTA. EDTA-treated samples were mixed with 10 mM Ca^2+^, Cd^2+^, Co^2+^, Mg^2+^, Mn^2+^, and Zn^2+^ (in the form of chloride salt) and incubated at room temperature for 20 min prior to assay. Esterase activity was measured using *p*-nitrophenyl acetate as described in [25] with some modifications. One milliliter of 25 mM Tris-HCl (pH 7.5) containing 20 µg of purified LCIP63 was used in the assay at 3 °C, and after 1.5 min the substrate was added (3 mM final concentration) to the mixture. The hydrolysis of *p-*nitrophenyl acetate was followed at 348 nm. A control was performed without the enzyme in the reaction to measure spontaneous hydrolysis. Production of *p-*nitrophenol was calculated using a molar extinction coefficient of 2457 M^−1^ cm^−1^. Proteins were measured using the Bradford assay with bovine serum albumin as a standard [26].

### Location of LCIP63 using immunogold labeling

Aliquots of *T*. *pseudonana* cells grown at 50 or 20,000 ppm (control) CO_2_ were centrifuged at 500 × *g* for 5 min. The pellet was pressure frozen, freeze substituted, and embedded in LR-White resin (Medium Grade), as reported in [27]. Ultrathin cryosections (60–90 nm) were cut with an ultracryomicrotome (EM UC7, Leica). The on-section immunolabelling was performed [27], with a primary antibody against TpLCIP63 (1:50 dilution) and protein A 10 nm as secondary antibody (Aurion), following by staining with uranyl acetate and lead citrate [28]. Samples were analyzed with a Tecnai G2 20 TWIN (200KV) transmission electron microscope (FEI) and digital images were acquired with a digital camera (Eagle, FEI). Before use, the specificity of the polyclonal antibodies of LCIP63 was checked against crude extracts of cells grown at low and high CO_2_ and one band was recognized corresponding to LCIP63, but only in the low-CO_2_ grown cells.

### LCIP63 overexpression

RNA was isolated from *T*. *pseudonana* grown as described above. Pellets from 50 ml cultures in exponential phase were resuspended in 3 ml of Trizol® (Invitrogen) and incubated for 5 min at room temperature. Two hundred microliters of chloroform per ml was added, vortexed vigorously and incubated for 2–3 min at room temperature. Tubes were centrifuged at 16,000 *g* for 15 min at 4 °C. Supernatants were collected and RNA was precipitated with isopropanol and resuspended in 50 µl of nuclease-free water. cDNA was synthesized using the RevertAid First Strand cDNA Synthesis Kit (Thermo Scientific™) following manufacturer's instructions. LCIP63 cDNA was amplified and cloned between the *tpfcp*8 promoter and terminator of a pTha-K1 vector [29] using the one-step sequence- and ligation-independent cloning method [30]. The forward and reverse primers were atatcgcat**c**cg*cggccg*catgaagttcacatccagct and accaatccagtatg*cggccg*ctcaaaccatcacagcttca, where underlined sequences correspond to the segment complementary with the vector and sequences in italics correspond to the *Not*I restriction enzyme site and a guanine nucleotide changed to a cytosine is shown in bold. *T*. *pseudonana* was transformed with this vector by cell bombardment using the biolistic PDS-1000/He particle delivery system (BIORAD, CA, USA) following a slightly modified protocol [31]. A total of 10^8^ cells in exponential phase were plated in F/2 + Si 1% agar plates before bombardment. Bombardment was performed twice with 300 µg of DNA-coated tungsten particles (0.5–1 µg of DNA) and cells were immediately transferred to liquid medium and kept for 24 h at 18 °C in low light, without shaking. Cells (10^6^–5 × 10^7^) were then transferred onto F/2 + Si 1% agar plates containing the antibiotic nourseothricin (Jena Bioscience) at a final concentration of 100 µg ml^−1^. Antibiotic resistant colonies were selected 3 weeks later and transferred to liquid medium.

### Measurement of photosynthesis versus DIC concentration

Three LCIP63-overexpressing clones and one clone transformed with the empty vector (control) were grown at 20,000 ppm CO_2_ for 72 h and then transferred to 50 ppm CO_2_ for another 24 h before harvesting. A total of 15 × 10^6^ cells were centrifuged at 3500 × *g* for 10 min at 18 °C, then washed twice with DIC-free artificial sea water containing 10 mM HEPES (pH 8). Changes in oxygen concentration of a suspension of 1 ml was measured at 240 µmol photon m^−2^ s^−1^ (400–700 nm) using a range of DIC concentrations in a Clark-type electrode (Oxygraph; Hansatech, Norfolk, UK) in triplicate [8]. Data were fitted to the Michaelis–Menten equation to estimate the maximal rate of net photosynthesis, $P_{net}^{max}$, and the concentration required for half maximal rate, *K*_0.5_ [DIC].

### Chlorophyll extraction and measurement

Two milliliters of cell culture was pelleted at 11,000 g for 10 min at 4 °C (Sigma® 2-16KC Centrifuge; Rotor 12132-H, Fisher Bioblock Scientific). The pellet was resuspended in 2 ml of ethanol 96% and incubated for 1 h at 4 °C in the dark. Samples were centrifuged at 11,000 *g* for 30 min at 4 °C. The optical density of the supernatant was measured at 629 and 665 nm and chlorophyll *a* calculated [32].

### Statistical analysis

Graph Pad Prism 6 software was used for all graphs and statistical analyses. Unpaired *t*-tests were used to determine whether two data sets were significantly different.

## Results

### Evidence that LCIP63 is a new CA subclass

**Fig. 1:**
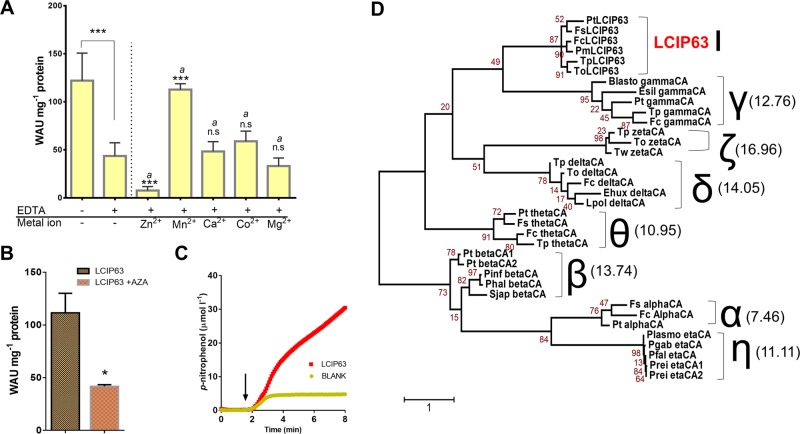
CA activity of purified recombinant LCIP63. **a** CA activity with and without treatment with 5 mM EDTA or EDTA plus 10 mM of either Zn^2+^, Mn^2+^, Ca^2+^, Co^2+^, or Mg^2+^. n.s, not significant; ***p ≤ 0.001. Error bars show mean ± SD, *n* = 3–19. ‘*a*’ indicates values compared to the EDTA-treated sample. **b** CA activity in LCIP63 treated with 20 µM acetazolamide (AZA) versus control; *p ≤ 0.05. **c** Esterase activity of LCIP63 expressed as the production of *p-*nitrophenol (µmol l^−1^). The arrow represents the time when the substrate *p*-nitrophenyl acetate was added to the reaction mixture. **d** Phylogenetic tree constructed from sequences of the different CA subclasses; numbers in parentheses are the mean percent identity with LCIP63. Pfal, *Plasmodium falciparum*; Prei, *Plasmodium reichenowi*; Plasmo, *Plasmodium* sp.; Pgab, *Plasmodium gaboni*; Pt, *Phaeodactylum tricornutum*; Fs, *Fistulifera solaris;* Fc, *Fragilariopsis cylindrus*; Tp, *Thalassiosira pseudonana*; Ehux, *Emiliania huxleyi*; Lpol, *Lingulodinium polyedra*; To, *Thalassiosira oceanica*; Esil, *Ectocarpus siliculosus*; Blasto, *Blastocystis* sp.; Pinf, *Phytophtora infestans*; Phal, *Plasmopara halstedii*; Sjap, *Saccharina japonica*; Tw, *Thalassiosira weissflogii*; Pm, *Pseudo-nitzschia multiseries*

The purified recombinant LCIP63 from *T*. *pseudonana* had a CA activity of about 122 ± 28 WAU mg^−1^ protein, with or without a histidine-tag (Figs. 1a and S1). All known CAs are metalloenzymes and when the CA activity of LCIP63 was measured after treatment with a metal ion-chelating agent, EDTA, it decreased significantly to 44 ± 13 WAU mg^−1^ protein (~64% reduction). Many CAs have Zn^2+^ as a cofactor, however the addition of Zn^2+^ to the EDTA-treated enzyme resulted in a complete loss of activity. The addition of cadmium or cobalt that can also act as cofactors for CAs from *T*. *weissflogii*, also did not restore CA activity and in the case of Cd^2+^ resulted in the total precipitation of LCIP63 preventing measurement (data not shown). Similarly, the addition of Mg^2+^ and Ca^2+^ to the EDTA-treated LCIP63 did not restore LCIP63 activity. In contrast, CA activity was fully restored by the addition of Mn^2+^ (Fig. 1a). In order to see whether LCIP63 can be redox regulated, we also measured CA activity in the presence of the reducing agent, dithiothreitol (DTT). We observed that CA activity is significantly decreased by 5 mM DTT treatment (equivalent to 10 mM thiol groups); however this inhibition is prevented in the presence of 20 mM Mn^+2^ (Fig. S2).

All CAs are inhibited by sulfonamides and the CA activity of LCIP63 was reduced by 74% after treatment with the CA inhibitor, acetazolamide at 20 µM (Fig. 1b). CA enzymes have promiscuous esterase activity that is a good indicator of CA activity [33] because the two reactions share a common mechanism and catalytic pocket. LCIP63 also had esterase activity when assayed with *p*-nitrophenyl acetate (Fig. 1c), that is consistent with its function as a CA.

Since LCIP63 behaved like a CA, we compared LCIP63 sequences from diatoms to sequences from the seven other known subclasses of CA. The identity between LCIP63 from *T*. *pseudonana* and CAs from different subclasses varied between 7% and 17% suggesting that LCIP63 is not closely related to any known CA (Fig. 1d), and so is a new CA subclass that we designated with the Greek letter iota (ι).

### Localization of LCIP63

**Fig. 2:**
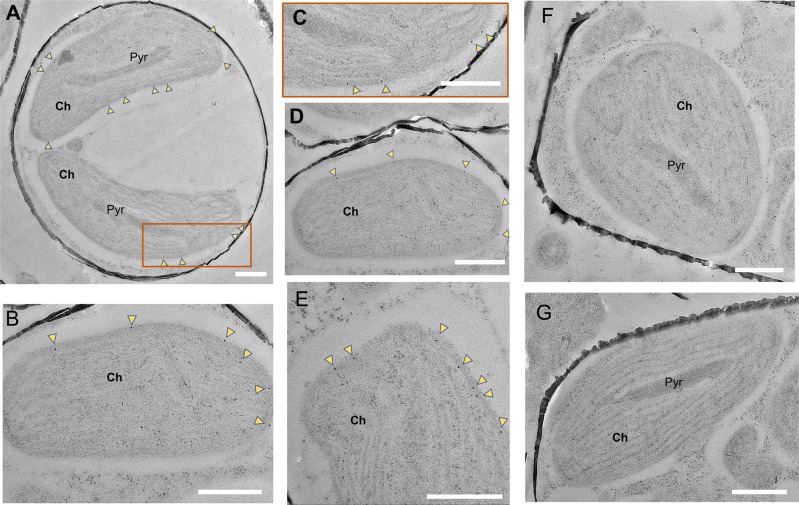
Transmission electron micrographs of immunogold labeled LCIP63 in *T*. *pseudonana*. **a–e** Cells cultured at 50 ppm CO_2_. **c** Close-up of the rectangular box in (**a**). **f**, **g** Cells cultured at 20,000 ppm CO_2_. Gold particles are indicated by yellow arrowheads. Ch, Chloroplast; Pyr, Pyrenoid. Bar = 500 nm

To confirm the predicted location of LCIP63 in the chloroplast based on the signal peptide [13], cells of *T*. *pseudonana* grown at low CO_2_ were immunogold labeled with specific antibodies against LCIP63. LCIP63 was clearly expressed in cells grown at low CO_2_ (Fig. 2a–e) and only found towards the chloroplast periphery. No expression was observed in cells grown at high CO_2_ that acted as a control (Fig. 2f, g). LCIP63 is thus the third known CA from the chloroplast of *T*. *pseudonana* [12].

### LCIP63 overexpression increases the affinity for dissolved inorganic carbon

**Fig. 3:**
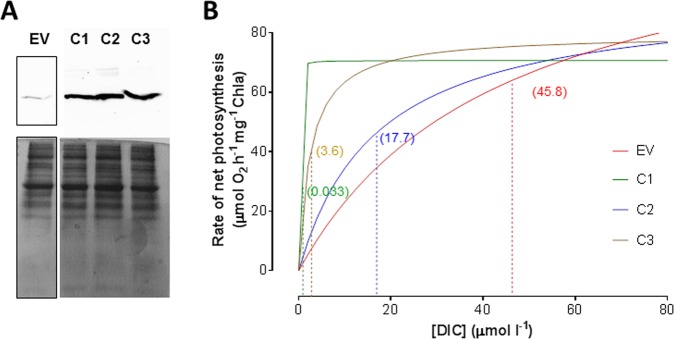
LCIP63 overexpression and effect on photosynthesis in *T*. *pseudonana*. **a** Western blot against LCIP63 (upper) and SDS-PAGE to verify protein loading (lower) for control cells and three LCIP63-overexpressing clones. **b** Rate of net photosynthesis versus concentration of DIC. EV corresponds to the clone transformed with an empty vector; C1, C2, C3, to clones 1, 2, and 3 overexpressing LCIP63. Theoretical curves showing a close-up of fits obtained with experimental data (3 replicates). Fits were performed using Michaelis–Menten equation; dashed vertical lines represent the mean of *K*_0.5_ [DIC] that are indicated in parentheses. All parameters and statistical analysis are given in Table 1

Western blots confirmed that LCIP63 was overexpressed in three clones compared to a control clone (Fig. 3a). At 20,000 ppm CO_2_, the growth rate was similar in all four clones (Fig. S3). The four clones were acclimated to 50 ppm CO_2_ for 24 h as previous work showed this induced LCIP63 expression [13]. The CO_2_-saturated rates of net photosynthesis ($P_{net}^{max}$) in the three overexpressing clones were not significantly different from the control even though the rate of the latter seems slightly higher than the other clones (Table 1). In contrast, although the *K*_0.5_ for dissolved inorganic carbon (DIC) differed among the three clones, in all cases it was significantly lower than that of the control (Fig. 3b, Table 1). This result shows that LCIP63 overexpression increases DIC affinity in photosynthesis, consistent with LCIP63 being an important component of the CCM. The *K*_0.5_ of the control clone was identical to that of cells grown at 20,000 ppm CO_2_, while that of two of the overexpressed clones were lower than that for wild-type cells grown at 400 or 50 ppm CO_2_ [8].

**Table 1:** Photosynthetic parameters of clones overexpressing LCIP63 compared to control cells, measured at pH 8

Cell	*P*_*net*_^*max*^ (µmol O_2_ h^−1^ mg^−1^ Chl*a*)	*K*_0.5_ [DIC] (µM)
EV	125.5 ± 35.8	45.8 ± 5.2
C1	71.2 ± 7.7^n.s., a^	0.033 ± 0.026^**, b^
C2	93.5 ± 8.4^n.s., a^	17.7 ± 3.9^**, b^
C3	79.2 ± 4.5^n.s., a^	3.6 ± 3.3^***, b^

All values are expressed as mean ± standard deviation (*n* = 3)

*EV* empty vector (control). C1, C2, and C3: LCIP63-overexpressing clones 1, 2, and 3, *P*_*net*_^*max*^ maximum photosynthetic rate, *K*_0.5_ [DIC]: DIC concentration to reach half of *P*_*net*_^*max*^ value

^n.s.^Not significant compared to control. **p ≤ 0.01, ***p ≤ 0.001

^a^Values compared to control *P*_*net*_^*max*^

^b^Values compared to control *K*_0.5_ [DIC]

### CA activity is affected by LCIP63 oligomeric state and not by the number of repeated domains

**Fig. 4:**
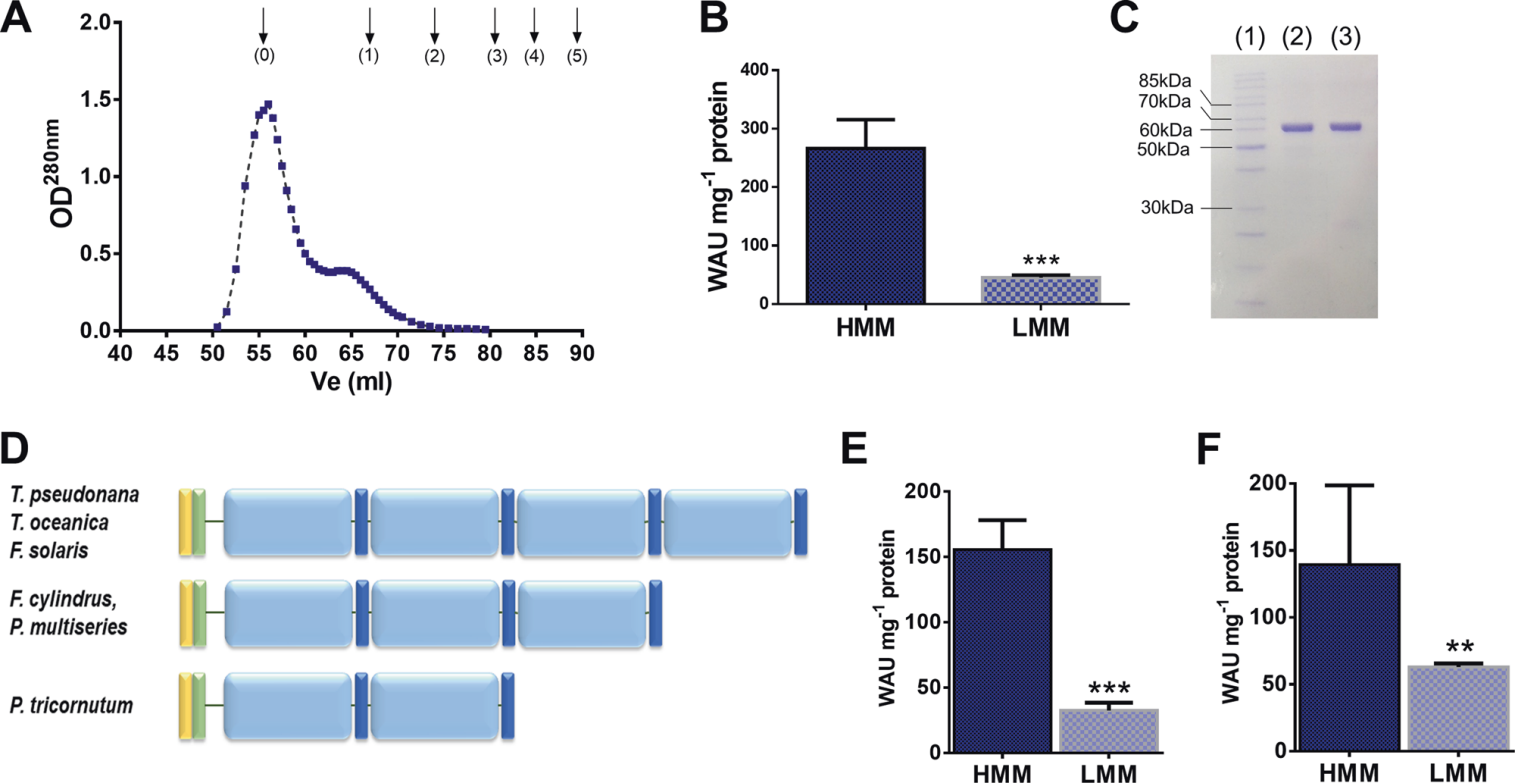
Characterization of two oligomeric forms of LCIP63. **a** Elution profile of LCIP63 using size-exclusion chromatography. Arrows indicate elution volumes of proteins used for calibration: 0, Void volume/Blue dextran (2000 kDa); 1, ferritin (440 kDa); 2, catalase (232 kDa); 3, glyceraldehyde-3-phosphate dehydrogenase (150 kDa); 4, bovine serum albumin (68 kDa); 5 ovalbumin (45 kDa). **b** CA activity of the high molecular mass form (HMM, eluted at 56 ml) and the low molecular mass form (LMM, eluted at 64 ml). **c** SDS-PAGE of LCIP63 (2 µg): (1) ladder, (2) HMM fraction, (3) LMM fraction. **d** Schematic representation of the primary structure of LCIP63 from six diatom species. Yellow and green boxes represent ER transit and chloroplast targeting peptides, respectively, light blue boxes represent CaMKII-AD and dark blue boxes the ‘(H)HHSS’ motif. **e, f** CA activity of the high molecular mass (HMM) and low molecular mass (LMM) forms of the LCIP63 variant containing three (**e**) and two (**f**) domain repeats. **p ≤ 0.01; ***p ≤ 0.001. Error bars show mean ± SD, *n* = 3–6

Size-exclusion chromatography showed that two forms of the purified recombinant LCIP63 were present. The more abundant, higher molecular mass (HMM) form was eluted in the void volume while the less abundant lower molecular mass form (LMM) had a molecular mass greater than that of ferritin at 440 kDa (Fig. 4a). The CA activity of the HMM form (266 ± 49 WAU mg^−1^) was higher than that of the LMM (45 ± 4 WAU mg^−1^; Fig. 4b). The presence of homogeneous LCIP63 in both fractions was confirmed by SDS-PAGE (Fig. 4c).

Using the LCIP63 sequence from *T*. *pseudonana*, sequences with an identity above 50% were retrieved in the five other diatom species with available sequenced genomes: *Thalassiosira oceanica*, *P*. *tricornutum*, *Fistulifera solaris*, *Fragilariopsis cylindrus*, and *Pseudo-nitzschia multiseries*. A four-repeat calcium-calmodulin protein kinase II association domain (CaMKII-AD), as previously observed in *T*. *pseudonana* LCIP63 [13], was found in *T*. *oceanica* and *F*. *solaris*, while the same domain was repeated three times in *F*. *cylindrus* and *P*. *multiseries* and two times in *P*. *tricornutum* (Fig. 4d). We constructed LCIP63 variants, based on the sequence of *T*. *pseudonana*, containing either three (LCIP63-3DOM) or two domain (LCIP63-2DOM) repeats. The monomers of these two proteins had a molecular mass of about 50 and 36 kDa, respectively, including the His-tag (2.3 kDa). As previously found for the four-domain LCIP63, the HMM and LMM forms were observed in both variants (Fig. S4), and again CA activity in the HMM form (153 ± 21 WAU mg^−1^ and 165 ± 53 WAU mg^−1^ for LCIP63-3DOM and LCIP63-2DOM, respectively) was higher than in the LMM form (33 ± 5 WAU mg^−1^ and 63 ± 3 WAU mg^−1^ for LCIP63-3DOM and LCIP63-2DOM; Fig. 4e, f).

### LCIP63 occurs in other organisms and is geographically widespread

**Fig. 5:**
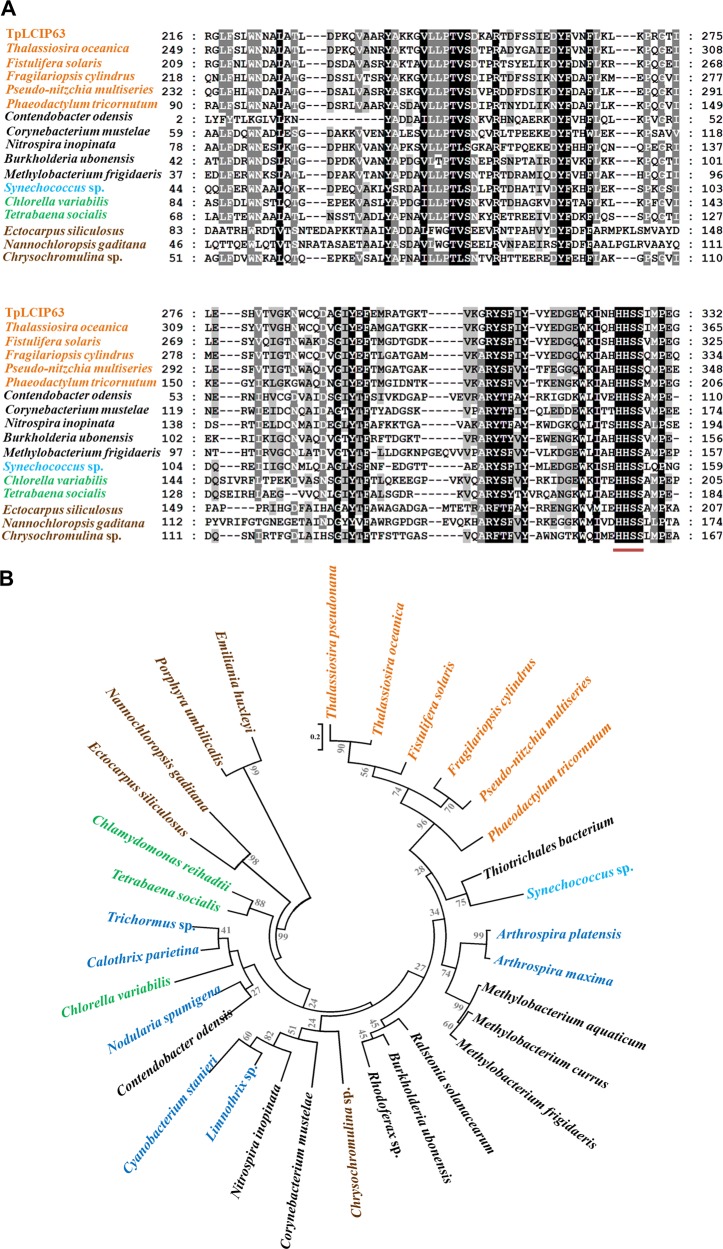
Alignment of the LCIP63 sequence from *T*. *pseudonana* and other organisms. **a** Alignment with the second LCIP63 domain was made with Mega6 software and analyzed in GeneDoc (http://www.psc.edu/biomed/genedoc). This domain has an identity of 60–68% with the other three LCIP63 domains and was the most similar to the sequences from the other species. The sequences with the highest score from diatoms (orange), bacteria (black), cyanobacteria (blue), green algae (green), and other Chromista algae (brown) are shown. Shading levels correspond to conserved amino acids: Black, 100% identity; dark gray, 80% identity; light gray, 60% identity. Complete alignment is given in Fig. S4. **b** Phylogenetic tree of proteins containing LCIP63-like domains. Bootstrap values are shown between nodes. The scale represents the number of substitutions per site. Colors are as in panel (**a**). More information about the sequences are in table S1

The results above suggest that all currently sequenced diatoms possess LCIP63 homologs that are functional CAs. In addition to the Bacillariophyceae, LCIP63 was also present, with a high similarity in Bangiophyceae, Chlorophyceae, Cyanophyceae, Eustigmatophyceae, Prymnesiophyceae, Phaeophyceae, and Trebouxiophyceae (Figs. 5a and S5) with an *E*-value ≤ 1.0 E^−24^. It was found with a high sequence score in many bacteria classes but not in animals or land plants. Since the sequence of LCIP63 was not recognized as a CA, all these proteins were annotated in NCBI either as having an unknown function or, for some prokaryote homologs, as belonging to the ‘SgcJ/EcaC oxidoreductases family’ (Table S1). A consensus “(H)HHSS” motif in all studied sequences was present at the C-terminus of each domain. A phylogenetic tree (Fig. 5b) showed that LCIP63 from diatoms forms one clade which is closer to bacterial and cyanobacterial sequences than to green algae or other algae from the chromista kingdom.

**Fig. 6:**
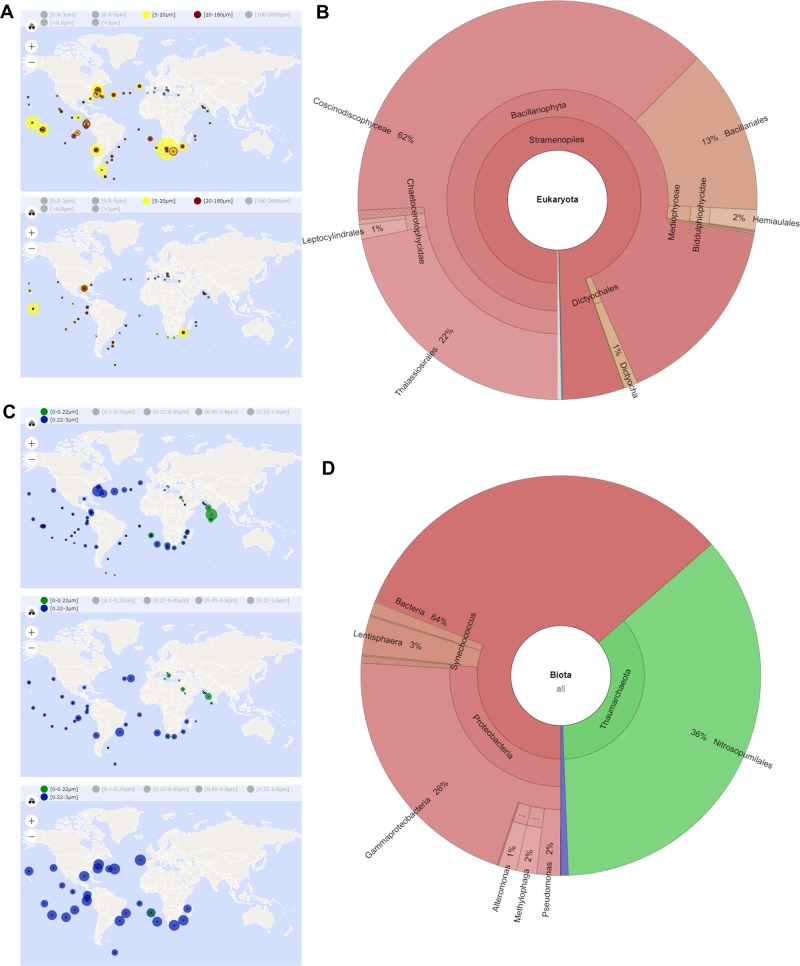
Geographic and taxonomic distribution of LCIP63 homolog sequences found in the OGA webserver. **a** Geographic distribution and abundance of LCIP63 homologs in eukaryotes in surface water (SRF; upper panel) and deep chlorophyll maximum layer (DCM; lower panel). **b** Taxonomic distribution of all the hits found in the OGA webserver for eukaryotes. **c** Geographic distribution and abundance of LCIP63 homologs in prokaryotes and picoeukaryotes in SRF (upper panel), DCM (middle panel), and mesopelagic zone (MES; lower panel). **d** Taxonomic distribution of all the hits found for prokaryotes and picoeukaryotes. The size of the filled circles shown in (**a**, **c**) are proportional to the abundance of the hits in one location compared to the total number of hits at the different sampled depths, and colors represent the size fractionation range

The complete protein sequence of LCIP63 was queried against the Tara Oceans database. All sampling sites contained LCIP63 sequences and using stringent *E*-values, 4924 hits were obtained for eukaryotes (*E*-value < 1.0 E^−30^) and 574 hits for prokaryotes (*E*-value < 1.0 E^−15^). Eukaryotic LCIP63 sequences were found predominantly in the surface water layer (SRF) and, less abundantly, in the deep chlorophyll maximum layer (DCM; Fig. 6a), and were more abundant south of the equator although this might result from seasonal patterns and sampling date. Prokaryotic LCIP63 sequences were almost equally distributed among the latitudes and sampled depths but, in contrast to eukaryotes, were more abundant in the mesopelagic zone (MES) compared to SRF and DCM (Fig. 6c).

Taxonomical distribution showed that almost 100% of all eukaryote hits corresponded to Stramenopiles (Fig. 6b) and 94% of these were Bacillariophyta. Using a threshold *E*-value of 1.0 E^−5^, a similar output was observed (not shown). There was also a small percentage of Alveolata (0.06%) and other eukaryotes (0.3%). Within the diatoms, the most abundant groups were Coscinodiscophyceae (67%), Thalassiosirales (24%), and Bacillariophyceae (14%). For prokaryotes, 36% of the hits were Achaea from the phylum Thaumarchaeota and 64% were bacteria (Fig. 6d), including the phylum Proteobacteria (27%), Lentisphaera (3%), Cyanobacteria (1%), and other unclassified bacteria (32%).

## Discussion

There is not necessarily a direct relationship between the primary sequence of a protein and its structure and function. Gan et al. [34] gave examples of proteins from nine different families that had overlapping 3D-structures but a sequence identity <20%. This is also the case for CA where there is a growing number of subclasses, currently seven [11], that have different sequences but catalyze the same hydratase reaction as well as a promiscuous esterase reaction. We show here that LCIP63 has a primary sequence that differs any of these CAs, and is not recognized as a CA in sequence databases. Nevertheless, the recombinant LCIP63 has CA activity in the range of 150–260 WAU mg^−1^ of protein, that is higher than the recombinant θ-CA from *P*. *tricornutum*, 42 WAU mg^−1^ of protein, but lower than the immunoprecipitated native form, 500 WAU mg^−1^ of protein [17]. In addition, LCIP63 has an esterase activity and is inhibited by the classic CA inhibitor acetazolamide; both are features of CA. Altogether, our data strongly suggest that LCIP63 belongs to a new subclass of CA that we designate as ι-CA.

All CAs require a metal ion for their catalysis that is most frequently zinc. However, the prokaryote *Methanosarcina thermophila*, contains a prototype γ-CA, where Fe^2+^ can substitute for Zn^2+^ [35]. Similarly, *T*. *weissflogii*, contains a cambialistic ζ-CA (CDCA1) that can use Zn^2+^ and Cd^2+^ [15, 36], and a δ-CA (TWCA1) that uses Zn^2+^ as well as Co^2+^ [37, 38]. Like other CAs, LCIP63 requires a metal ion to function, since treatment with EDTA, significantly reduced its activity. Surprisingly, the addition of Zn^2+^ to EDTA-treated LCIP63 suppressed CA activity further, while its activity was fully recovered by Mn^2+^, an essential trace metal for marine phytoplankton [39–42]. Submission of the four-domain LCIP63 sequence to the IonCom website (https://zhanglab.ccmb.med.umich.edu/IonCom/) [43] resulted in the identification of Mn^2+^ binding site at Histidine 269, Glutamate 281, Aspartate 462, and Histidine 525. In contrast, IonCom predicts many Zn^2+^ binding residues and therefore Zn^2+^ might bind aspecifically to LCIP63 resulting in the inhibition of its activity, as we observed.

Several lines of evidence suggest that an Mn-based CA is feasible. Mn^2+^ can replace Zn^2+^ in bovine β-CA [44], and in *T*. *pseudonana*, the superoxide dismutase is based on Mn^2+^, in contrast to many other species [45]. Ecologically, Mn^2+^ is highly available since total dissolved inorganic concentrations range from about 2 nM at the surface of open oceans to over 500 nM near the mouth of estuaries [46]. These concentrations are 20- to 50-times higher than those of zinc in estuaries, and the ocean where zinc concentrations can fall below 50 pM [47]. *T*. *pseudonana* is a coastal species and its *K*_0.5_ for (total dissolved inorganic) Mn^2+^ uptake is about 80 nM, but growth rates are saturated with Mn^2+^ as low as 10 nM [46] so, under natural conditions, Mn^2+^ concentrations will be close to saturating. The oceanic *T*. *oceanica*, that also has LCIP63, has a *K*_0.5_ for Mn^2+^ uptake that is sevenfold lower than that of *T*. *pseudonana* and so is also likely to be close to Mn^2+^ saturation even at low Mn^2+^ concentrations [46]. The average of Mn:Zn ratio in phytoplankton from the green lineage was 4.4, but 9.7 from the red lineage (Fig. S1 in [48]), that may reflect a greater reliance on Mn for this lineage. The cadmium-containing CDCA1 from *T*. *weissflogii*, is also found in *T*. *pseudonana* (XP_002295227.1) as well as the cobalt-containing TWCA1 (XP_002287620.1), so with LCIP63, *T*. *pseudonana* can potentially rely on Mn^2+^, Cd^2+^, and Zn^2+^ to maintain CA activity.

Several β-CA from different organisms are shown to be redox regulated [49–51]. Here we observed that although LCIP63 is affected by DTT treatment, this might not be related to redox regulation but to the chelation of the metal ion by the thiol groups of DTT since the effect of DTT is abolished by high concentrations of Mn^+2^.

Most CAs are monomeric although β-CAs can form dimers, tetramers, or octamers [14] and γ-CAs, trimers [52]. LCIP63 contains a four-repeat CaMKII-AD that in other proteins is responsible for the assembly of 8–14 subunits into large multimers [53]. Consistent with this, LCIP63 exists in two multimeric forms, both with a high molecular mass. These two forms have different activities as do the different forms of β-CA from both *E. coli* and *Mycobacterium tuberculosis* [54, 55].

LCIP63 from the six diatom genomes that are available had CaMKII-AD domains that varied between two and four, but all had CA activity. Repeat domains are also present in the Cd-containing CA (CDCA1-R1, -R2, and -R3) from *T*. *weissflogii* [15]. LCIP63-like proteins containing two domains were present in some Chlorophyceae and *P*. *tricornutum* and, by analogy to the *T*. *pseudonana* LCIP63 variant with two domain repeats, these proteins might also have CA activity.

LCIP63 contains a chloroplast signal peptide, an endoplasmic reticulum transit peptide and, unlike the θ-CA from *P*. *tricornutum*, does not possess the thylakoid-targeting domain [17], and so was expected to occur within the stroma [56]. Concordantly, electron microscopy showed that it is located close to the inner chloroplast membrane surrounding the stroma. There are no predicted transmembrane domains in LCIP63, so it is unlikely to be anchored to the chloroplast inner membrane by itself but LCIP63 may be a peripheral membrane protein interacting with a membrane component, but this needs to be tested.

CAs play an important role in the CCMs of diatoms and photosynthetic organisms [9, 57, 58]. CA inhibitors reduce the affinity for DIC in *P*. *tricornutum* [59] and *T*. *pseudonana* [8]. When the α-CA, from the eustigmatophyte *Nannochloropsis oceanica* was mutated, the affinity for DIC was drastically reduced in cells acclimated to low CO_2_ [60]. The affinity for DIC was also affected when θ-CA from *P*. *tricornutum* was overexpressed or silenced [17]. We found that overexpression of LCIP63 dramatically increased the affinity for DIC, strongly suggesting that LCIP63 is essential for the diatom CCM when cells are acclimated to low CO_2_.

Sequences with homology to LCIP63 in bacteria are frequently annotated as a putative SgcJ/EcaC family oxidoreductase and further work is required to determine if they have CA activity. These sequences only have one domain apart from the only Gram-positive bacterium recovered, *Corynebacterium mustelae,* and the gram-negative *Contendobacter odensis*, where two were present. Although proteins with repeat domains are present throughout the tree of life and are believed to be the consequence of duplication of genes or segments inside one gene, they are much less abundant in prokaryotes than in eukaryotes [61], in agreement with our findings. Diatoms contain genes from different origins including archaea and bacteria [4, 18] and LCIP63 may therefore derive from the duplication of a prokaryotic gene. Each domain from putative LCIP63 proteins contains a (H)HHSS conserved motif that is not present in other NTF2-like superfamily domain-containing proteins, nor in the conserved CaMKII(AD). This motif is therefore a specific feature of this protein. Since histidine residues are often found in the metal binding pocket of CA [11], one might expect this highly conserved motif to be involved in the metal ion binding of LCIP63; and two histidine residues H269 and H525 predicted by IonCom belong to this motif. Data from the OGA webserver showed that LCIP63 is widely present among marine planktonic organisms, including bacteria and archaea.

In eukaryotes, LCIP63 is found only in water layers close to the surface (SRF and DCM) probably because at greater depths, i.e., mesopelagic zone (MES; 200–1000 m deep), there is less light availability and therefore fewer photoautotrophs [62]. In contrast, LCIP63 homolog sequences from Bacteria and Archaea were more abundant (yet not exclusive) in the MES, a zone of organic matter decomposition [62]. The most abundant prokaryotes containing LCIP63 included gammaproteobacteria and the archaeal phylum Thaumarchaeota, both being ammonia-oxidizing microorganisms [63]. The precise role of LCIP63 in nonphotosynthetic organisms awaits discovery.

The large and increasing number of CA families without an apparent common ancestor and their ubiquitous presence in all domains of life demonstrates the biological importance of this enzyme. LCIP63 is found in all available sequences from marine diatoms and thus might be ubiquitous in the Bacillariophyceae. LCIP63 is also present in sequenced eukaryotic algae from seven classes, Cyanobacteria, bacteria, and archaea. As more sequences become available it seems likely that LCIP63 will be found in a greater number of organisms and research should include freshwater plankton that are currently understudied. With a possible prokaryote origin, LCIP63 has evolved to become a protein that seems to be important for aquatic photosynthesis. It appears to use manganese as a cofactor, reinforcing the potential of marine diatoms to adapt to the availability of trace metals in their environments. More work is needed to establish if Mn^2+^ is the metal cofactor in the native CA from *T*. *pseudonana* and determine if transcriptional regulation by metal availability occurs as is the case for some cambialistic CAs. More biochemical and biophysical characterization of this new widespread subclass of CA, ι-CA, should be undertaken to understand better its role in global biogeochemical cycling.

## Supplementary information

Supplementary figures

Table S1
