# Supplementary material for: A new widespread subclass of carbonic anhydrase in marine phytoplankton
Source: ISME J. 2019 Apr 25;13(8):2094–106. doi: 10.1038/s41396-019-0426-8 (PMC6776030; doi:10.1038/s41396-019-0426-8)
Supplement: Supplementary file 1 — Supplementary figures [file 41396_2019_426_MOESM1_ESM.docx]

**SUPPORTING INFORMATION**


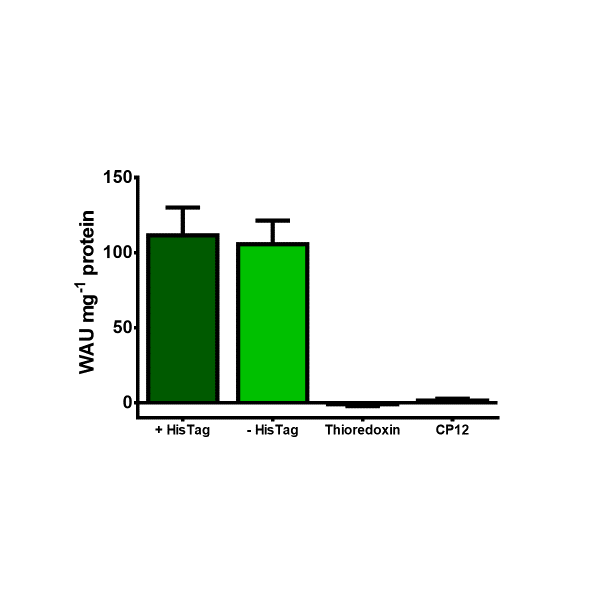


**Fig. S1.** CA activity of controls. LCIP63 CA activity was compared in the recombinant protein containing the HisTag or after HisTag removal by thrombin, and after treatment with acetazolamide (AZA; 20 µM). CA activity in HisTagged-thioredoxin and HisTagged-CP12 was also measured. Error bars show SD, n=3.


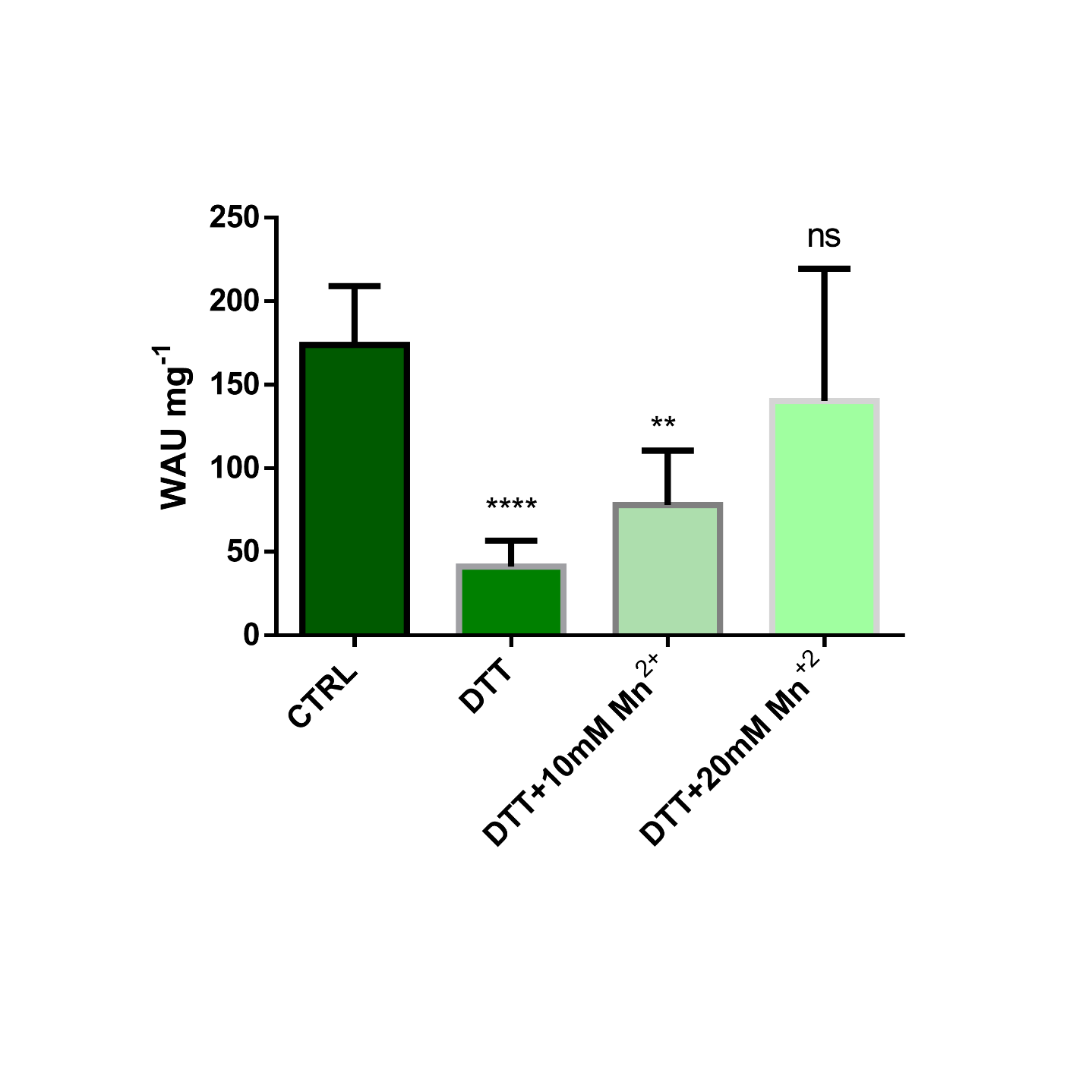


**Fig. S2.** CA activity in the presence of DTT (5 mM) and manganese. CA activity of LCIP63 was measured after treatment for 20 min. on ice, with 5 mM DTT alone (DTT) or in the presence of DTT and 10 or 20 mM Mn^+2^. All treatments are compared to control (CTRL). **** p<0.001, ** p<0.01, ns not significant. Error bars show SD, n= 4-9.


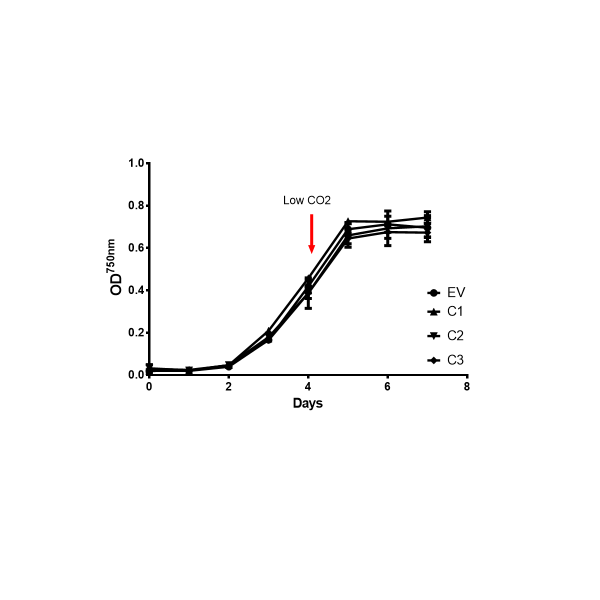


**Fig. S3.** Growth of *T. pseudonana* clones overexpressing LCIP63. Cultures were maintained at 20 000 ppm (high CO_2_) during the first four days and then transferred to 50 ppm (Low CO_2_; red arrow). EV: cells transformed with the empty vector. C1, C2, C3: Independent clones transformed with the LCIP63 overexpressing vector. Error bars show SD, n=3.


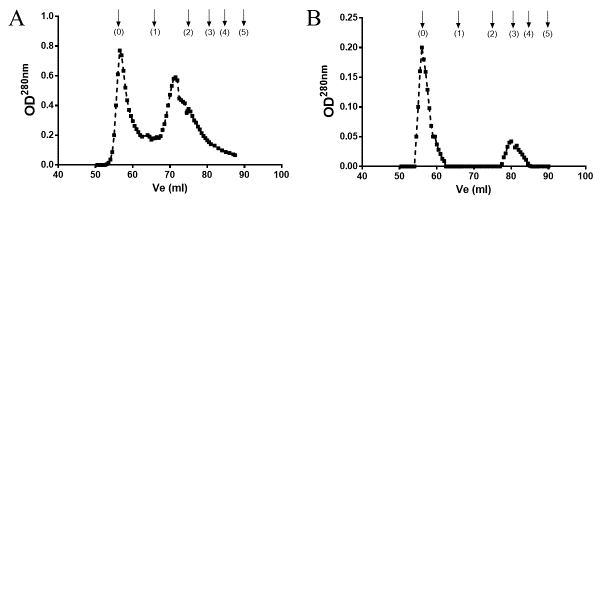


**Fig. S4.** Size exclusion chromatography profile of LCIP63 variants containing three (A) and two (B) domain repeats. The first and second peaks correspond to HMM and LMM. Arrows indicate elution volumes of proteins used for calibration: (0), Void volume/Thyroglobulin (660 kDa); (1), Ferritin (440 kDa); (2), Catalase (232 kDa); (3), Glyceraldehyde-3-phosphate dehydrogenase (150 kDa); (4), Bovine serum albumin (68 kDa); (5), Ovalbumin (45 kDa).


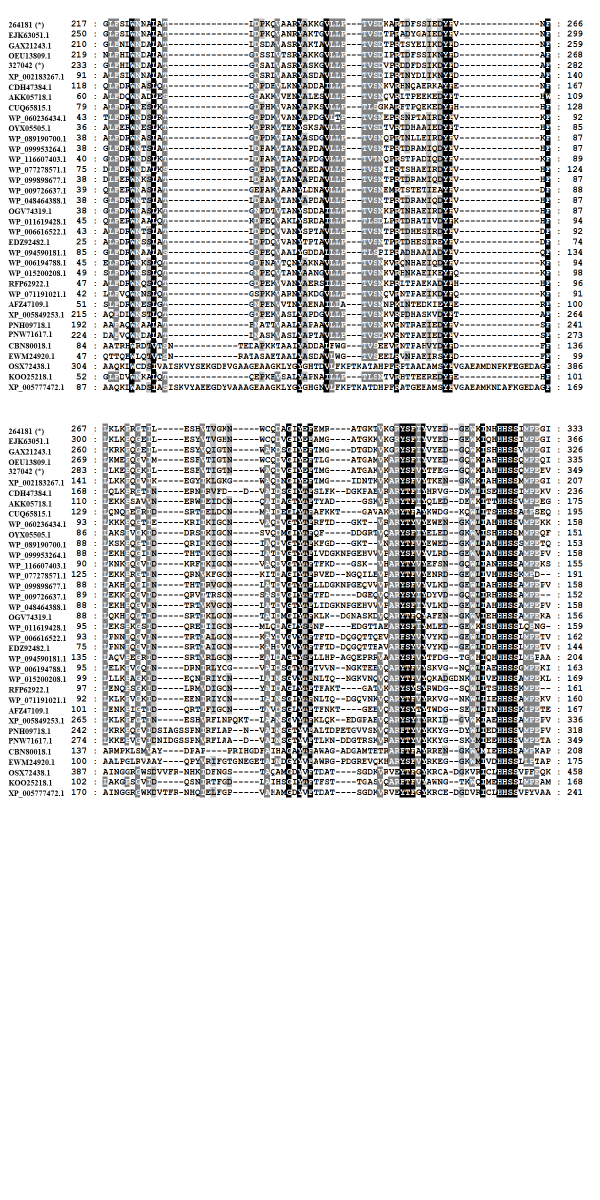


**Fig. S5.** Alignment of hypothetical LCIP63 sequences from different organisms with a maximum score greater than 100. Shading levels correspond to conserved amino acids: Black, 100% identity; dark grey, 80% identity; light grey, 60% identity. Accession numbers and details of the proteins are shown in Table S1.
