# Supplementary material for: A new widespread subclass of carbonic anhydrase in marine phytoplankton
Source: ISME J. 2019 Apr 25;13(8):2094–106. doi: 10.1038/s41396-019-0426-8 (PMC6776030; doi:10.1038/s41396-019-0426-8)
Supplement: Supplementary file 2 — Table S1 [file 41396_2019_426_MOESM2_ESM.docx]

| **Table S1. List of sequences used for alignment.** | | | |
| --- | --- | --- | --- |
| **Seq ID / Acc. Num.** | **Protein name** | **Species** | **Class** |
| 264181 *† | Hypothetical protein THAPSDRAFT_264181, partial | *Thalassiosira pseudonana* | Bacillariophyceae |
| EJK63051.1 † | Hypothetical protein THAOC_16311 | *Thalassiosira oceanica* | Bacillariophyceae |
| GAX21243.1 † | Hypothetical protein FisN_1Lh071 | *Fistulifera solaris* | Bacillariophyceae |
| OEU13809.1 † | Hypothetical protein FRACYDRAFT_269935 | *Fragilariopsis cylidrus* | Bacillariophyceae |
| 327042 *† | estExt_fgenesh1_pg.C_5280005 | *Pseudo-nitzchia multiseries* | Bacillariophyceae |
| XP_002183267.1† | Predicted protein | *Phaeodactylum tricornutum* | Bacillariophyceae |
| CDH47384.1 † | Conserved hypothetical protein | *Candidatus Contendobacter odensis* | Gammaproteobacteria |
| AKK05718.1 † | Hypothetical protein CMUST_06920 | *Corynebacterium mustelae* | Actinobacteria |
| CUQ65815.1 † | Conserved protein of unknown function | *Candidatus Nitrospira inopinata* | Nitrospira |
| WP_060236434.1 † | SgcJ/EcaC family oxidoreductase | *Burkholderia ubonensis* | Betaproteobacteria |
| OYX05505.1 | DUF4440 domain-containing protein | *Thiotrichales bacterium* | Gammaproteobacteria |
| WP_089190700.1 | SgcJ/EcaC family oxidoreductase | *Ralstonia solanacearum* | Betaproteobacteria |
| WP_099953264.1 | SgcJ/EcaC family oxidoreductase | *Methylobacterium currus* | Gammaproteobacteria |
| WP_116607403.1 | SgcJ/EcaC family oxidoreductase | *Rhodoferax sp.* | Betaproteobacteria |
| WP_077278571.1 | SgcJ/EcaC family oxidoreductase | *Thioalkalivibrio denitrificans* | Gammaproteobacteria |
| WP_099898677.1 † | SgcJ/EcaC family oxidoreductase | *Methylobacterium frigidaeris* | Alphaproteobacteria |
| WP_009726637.1 | SgcJ/EcaC family oxidoreductase | *Methylophaga lonarensis* | Gammaproteobacteria |
| WP_048464388.1 | SgcJ/EcaC family oxidoreductase | *Methylobacterium aquaticum* | Alphaproteobacteria |
| OGV74319.1 | DUF4440 domain-containing protein | *Methylophilales bacterium* | Betaproteobacteria |
| WP_011619428.1 † | SgcJ/EcaC family oxidoreductase | *Synechococcus sp.* | Cyanophyceae |
| WP_006616522.1 | SgcJ/EcaC family oxidoreductase | *Arthrospira platensis* | Cyanophyceae |
| EDZ92482.1 | Calcium/calmodulin dependent protein kinase II association-domain protein | *Arthrospira maxima* | Cyanophyceae |
| WP_094590181.1 | SgcJ/EcaC family oxidoreductase | *Vulcanococcus limneticus* | Cyanophyceae |
| WP_006194788.1 | SgcJ/EcaC family oxidoreductase | *Nodularia spumigena* | Cyanophyceae |
| WP_015200208.1 | SgcJ/EcaC family oxidoreductase | *Calothrix parietina* | Cyanophyceae |
| RFP62922.1 | SgcJ/EcaC family oxidoreductase | *Limnothrix sp.* | Cyanophyceae |
| WP_071191021.1 | SgcJ/EcaC family oxidoreductase | *Trichormus sp.* | Cyanophyceae |
| AFZ47109.1 | Calcium/calmodulin dependent protein kinase II association-domain protein | *Cyanobacterium stanieri* | Cyanophyceae |
| XP_005849253.1 † | Hypothetical protein CHLNCDRAFT_143501 | *Chlorella variabilis* | Trebouxiophyceae |
| PNH09718.1 † | Hypothetical protein TSOC_003635 | *Tetrabaena socialis* | Chlorophyceae |
| PNW71617.1 | Hypothetical protein CHLRE_16g661750v5 | *Chlamydomonas reinhardtii* | Chlorophyceae |
| CBN80018.1 † | Similar to Uncharacterized protein conserved in bacteria with a cystatin-like fold | *Ectocarpus siliculosus* | Phaeophyceae |
| EWM24920.1 † | Uncharacterized protein Naga_100092g2 | *Nannochloropsis gaditana* | Eustigmatophyceae |
| OSX72438.1 | Hypothetical protein BU14_0434s0001 | *Porphyra umbilicalis* | Bangiophyceae |
| KOO25218.1 † | Calcium/calmodulin dependent protein kinase II association-domain protein | *Chrysochromulina sp.* | Prymnesiophyceae |
| XP_005777472.1 | Hypothetical protein EMIHUDRAFT_443720 | *Emiliania huxleyi* | Prymnesiophyceae |
| *ID from JGI database. |  |  |  |
| †Sequences used for alignment in Fig. 5 and S4 | |  |  |
